# Sex and spatial proximity affect ungulate behavioral synchrony

**DOI:** 10.7717/peerj.21331

**Published:** 2026-05-20

**Authors:** George M.W. Hodgson, Kate J. Flay, Tania A. Perroux, Alan G. McElligott

**Affiliations:** 1 Department of Infectious Diseases and Public Health, Jockey Club College of Veterinary Medicine and Life Sciences, City University of Hong Kong, Hong Kong, China; 2 Centre for Animal Health and Welfare, Jockey Club College of Veterinary Medicine and Life Sciences, City University of Hong Kong, Hong Kong, China; 3 Department of Veterinary Clinical Sciences, Jockey Club College of Veterinary Medicine and Life Sciences, City University of Hong Kong, Hong Kong, China

**Keywords:** *Bos taurus*, Cohesion, Dominance, Foraging, Group coordination, Ruminant, Sexual segregation

## Abstract

Collective group decisions are important for the survival and reproduction of social mammals, with inter-individual interactions affecting group-level emergent behavior. Activity synchronization is an important aspect of collective behavior, with differences in nutritional requirements leading to foraging asynchrony. Individual variation between animals (such as sex or social relationships) are predicted to affect ungulate synchronization and spatial proximity, with between-sex differences consequently expected to influence the evolution of sexual segregation in ungulates. Although investigated independently, the relative roles of sex, sociality and proximity in synchronization are rarely investigated concurrently, especially in regards to affiliative relationships. Using a mixed-sex group of feral cattle (*Bos taurus*), we evaluated the supporting evidence for several predictions arising from the current understanding of synchronization in ungulates. We investigated how sex and social relationships (dominance and affiliation) affected foraging, behavioral synchrony and proximity. We found that same-sex dyads were more likely to be synchronized than mixed-sex dyads, while differences in dominance and affiliation only affected dyadic synchrony when observations with high foraging activity were excluded. Focal animals were more synchronized with closest neighbors than with another randomly selected conspecific. Inter-individual differences can explain variation in activity, with synchronization being biased towards certain individuals by favoring animals in close spatial proximity and those of the same-sex.

## Introduction

Behavioral synchronization plays a key role in collective movement and is widespread across taxa and behaviors (Schweinfurth, Stieger & Taborsky, 2017; Evans, Lihou & Rands, 2018; Maeda et al., 2021). In the present study, we define synchrony in activity as occurring when two or more individuals in the same location conduct the same action simultaneously (Asher & Collins, 2012; Duranton & Gaunet, 2016). Several definitions and potential mechanisms have been proposed to explain underlying synchronous behavior, which can arise from coupling mechanisms between individuals (for a more detailed discussion on possible synchrony mechanisms and self-organization, see Camazine et al., 2020; Greenfield et al., 2021; Hoehl, Fairhurst & Schirmer, 2021). By maintaining group cohesion and helping individuals stay in close proximity, synchrony in activity can provide considerable functional benefits (Boinski, 1987; Pays et al., 2007; Prokopenko et al., 2024), including effective foraging, enhancement of vigilance and deterrence of predators (Jackson, Ruxton & Houston, 2008; Tarr, Launay & Dunbar, 2016). However, animals often have to differentiate between activities of varying costs and benefits; individuals may face consensus costs if their interests do not align with the majority of their peers and may be forced to forfeit the advantages of social living if they deviate from the group consensus (King et al., 2008; Kerth, 2010; Sueur & Deneubourg, 2011). Synchronous behavior can affect group-level emergent properties (Ioannou & Laskowski, 2023), with activity and synchronization expected to be affected by both internal state (*e.g.*, motivation, nutritional requirements) and external influences (*e.g.*, food resources, presence of nearby group members, predation pressure; Asher & Collins, 2012).

Differences in individual physiology (such as those related to sex) are predicted to affect individual activity budget and time spent foraging, and therefore the likelihood of synchrony and proximity between two animals (Michelena et al., 2004; Šárová, Špinka & Panamá, 2007; Bowyer et al., 2020). Synchrony may be costly to individuals if it requires them to make a choice between staying synchronized and a non-synchronized action with a higher benefit to themselves, as not all animals benefit equally from performing the same behavior (Ruckstuhl, 1998). This trade-off is often greater when individuals are of different ages, sexes or reproductive status with different nutritional requirements; for example, sexually dimorphic ungulate males and females differ in rumen size and nutrient requirements (Bowyer et al., 2020). Males therefore eat lower quality forage but require a greater amount than females, leading to the sexes being unable to synchronize their activity (Barboza & Bowyer, 2000; Mooring et al., 2005). Asynchrony between individuals of different characteristics can result in fluctuating group membership and may play a role in segregation, but sex, synchrony and proximity have rarely been examined concurrently (Ruckstuhl & Neuhaus, 2002; Bonenfant et al., 2004; Mooring et al., 2005).

Spatial proximity to other animals affects synchrony, coordination and the spread of information throughout a group, but there is relatively little information on how social relationships can mediate this effect (Amichay et al., 2024). Synchronization through close proximity can be mediated through social facilitation (where the presence or behavior of an animal can directly or indirectly influence another individual’s activity) and individuals may synchronize their activity with the animals closest to them (Rands, Muir & Terry, 2014; Evans, Lihou & Rands, 2018; Hoyle, Miller & Rands, 2021). Behavior events can be unintentionally contagious, with nonconscious behavioral mimicry influencing individuals to change their activity to become similar to that of social partners within view; this social contagion can thus lead to the synchronization of all individuals in a group (Lakin et al., 2003; McDougall & Ruckstuhl, 2018; Ostner, Wilken & Schülke, 2021). Proximity and synchronization may also be unintentionally influenced by environmental cues such as foraging quality (Asher & Collins, 2012). However, individuals may mediate their responses to nearest neighbors depending on their identity, and the extent of the impact of social status on synchrony remains unknown (McDougall & Ruckstuhl, 2018; Valente et al., 2022), as synchronization is rarely examined concurrently relative to the characteristics of nearby social partners and their physical proximity.

Individual variation in social phenotype (*e.g.*, dominance rank and social bonds) is likely to affect animal movement, proximity and synchronization (Connor, Smolker & Bejder, 2006; Krueger et al., 2014; Fele et al., 2025). Dominance can affect foraging activity, with higher-ranked individuals monopolizing higher-quality foraging sites and forcing lower-ranked animals into areas of lower quality forage or higher predation risk (Schneider, 1984; Waite, 1987). Subordinate animals may experience socially mediated interference while foraging, occurring when a dominant individual reduces another animal’s energy intake and leading to subordinates having lower energy reserves (Rands et al., 2006). Social relationships (*e.g.*, affiliation or familiarity) affect the intensity of synchronization (Gygax, Neisen & Wechsler, 2010; Maeda et al., 2021), the likelihood of proximity (Briard, Dorn & Petit, 2015; Keshavarzi et al., 2023) and behavioral contagion (Valente et al., 2022), but it is often difficult to determine affiliative relationships in wild ungulates.

Cattle (*Bos taurus*) are highly social, sexually dimorphic mammals, and engage in both affiliative and agonistic behavior. With clear dominance hierarchies and stable social relationships, cattle present an excellent opportunity to investigate how individual characteristics affect behavioral synchrony in a globally abundant ungulate (Bouissou et al., 2001; Polák & Frynta, 2010). In beef and dairy cattle, behavioral synchronization is related to time, proximity and similarity in body size, but not dominance status or reproductive state (Šárová, Špinka & Panamá, 2007; Stoye, Porter & Stamp Dawkins, 2012). With the majority of cattle research performed on cattle in farms, previous studies often examine synchronization from a productivity and animal welfare viewpoint. However, farm cattle social organization and space use is affected by husbandry, with food delivery times and movement restrictions affecting social interactions and food competition (Bouissou et al., 2001). This in turn can artificially synchronize activity, with farm cattle synchrony being affected by space availability (Nielsen et al., 1997; Færevik et al., 2008), group size (Schneider et al., 2020), and environment (Krohn, Munksgaard & Jonasen, 1992; Tuomisto et al., 2019).

In Hong Kong, cattle were once used as draught animals until the decline of agriculture led to their release into surrounding areas during the 1950s–1970s (Barbato et al., 2020; Agriculture, Fisheries and Conservation Department of Hong Kong (AFCD), 2024). Since then, animals are free-ranging in certain districts of Hong Kong with limited routine husbandry (Dudgeon & Corlett, 1994; Perroux et al., 2025a). Nowadays, free-ranging feral cattle may live together for many years with stable dominance hierarchies and variation in social bond strength (Hodgson et al., 2025b; Hodgson et al., 2025c). Although Hong Kong feral cattle are sexually dimorphic in body size (with males being larger than females), this dimorphism is moderate compared to other large ungulates, including most cattle breeds (Perroux et al., 2025b).

In the present study, we examined the relationships between dyadic sociodemographic factors, spatial proximity and behavioral synchronization, investigating several hypotheses arising from our current understanding of synchronization in ungulates. Specifically, as differences in individual physiology and social status are expected to affect activity budget, we hypothesized that sex and dominance rank would affect foraging activity. We predicted (i) that males and higher-ranking animals spend less time foraging than females and lower-ranking animals (Ruckstuhl & Neuhaus, 2002). Second, we hypothesized that animals would synchronize their activity with their nearest neighbors, predicting (ii) that spatial proximity affected the likelihood of behavioral synchronization (Rands, Muir & Terry, 2014; Hoyle, Miller & Rands, 2021). Finally, we hypothesized that sociodemographic factors affected synchrony and proximity; we predicted (iii) that similarities in sex, affiliation and dominance affected the likelihood of synchrony and close proximity between a dyad, with dyads composed of the same sex, of similar rank, or having previously exchanged affiliation being more likely to be synchronized and in close proximity (Conradt, 1998; Connor, Smolker & Bejder, 2006). Examining these factors concurrently allows a unique investigation into the driving forces of synchronization in ungulates.

## Materials & Methods

### Ethical statement

This work was approved by the Animal Research Ethics Sub-Committee of City University of Hong Kong (Internal Reference: A-0826). This was an observational study, with animals remaining in their natural environment throughout and after observations. Feral cattle were observed without any human intervention, and disturbance to the animals was minimized in accordance with the ASAB/ABS ethical guidelines (ASAB Ethical Committee/ABS Animal Care Committee, 2023). Portions of this text were previously published as part of a preprint (Hodgson et al., 2025d).

### Data accessibility statement

Data with code are accessible *via* an online OSF repository (Hodgson et al., 2025a).

### Study area and subjects

We collected observational data on a mixed-sex group (herd) of feral cattle, located in Kuk Po, Plover Cove, in the northeastern New Territories of Hong Kong SAR, China. Cattle were observed throughout the study period in an area of approximately 0.28 km^2^ (22°31′46″N 114°14′05″E), with surrounding areas consisting of brackish wetland, mangroves, small village houses, woodland and coastland. Several hiking trails run through the study site, with cattle habituated to human presence. There were 18 individuals observed at the study site over the study period (13 females, five males). This group was selected due to their known social relationships, high location fidelity, and because cattle did not receive regular organized provisioning with supplementary food; this group is also representative of the mixed-sex social structure and average herd size relative to other Hong Kong populations (Hodgson et al., 2025b; Perroux et al., 2025b). One male was observed in the group for a maximum of 9 min on one day only; this male and any observations during this period were removed from the dataset to avoid any potential bias towards rarely seen animals, resulting in 17 animals used in data analysis. Individual cattle age was unknown but only cattle over 1 year in age (identified by emerged horns and comparable size to known individuals) were observed in the group over the study period. The Agriculture, Fisheries and Conservation Department of Hong Kong (AFCD) uses routine cattle sterilization (for both males and females) for mitigating human-animal conflict and for population management (Agriculture, Fisheries and Conservation Department of Hong Kong, AFCD)(2024). A vaccination program from 2016 to 2019 monitored reproduction in female cattle treated with a GnRh-based immunocontraceptive (Massei et al., 2015; Pinkham et al., 2022). However, there is no evidence of continued vaccination or that the ear-tagged identified cattle in that program were the same as the ones in this present study. Approximately 62% of males in the Hong Kong feral cattle population were sterilized by 2022 (Pinkham et al., 2022). Variation in male castration method can create high uncertainty when visually assessing reproductive status. However, we were unable to see any clearly visible signs of male castration within our study group, with bulls having visibly intact scrotums, although scrotal contents could not be palpated. Although we cannot confirm paternity, we observed two calves born to separate mothers within the 6 months prior (circa September 2022) from the date of this study (Hodgson et al., 2025b). These two calves were no longer present in the group by the observation date. Although the exact sterilization and reproductive status of the study animals were unknown, the presence of calves in this group indicates breeding activity with multiple intact females. While estrus-related behaviors have previously been observed in this group at this location, no clear estrus or reproductive behaviors were observed during the observation period. The mean observed group size during observations was 13.61 (range 8–17, standard deviation (SD) ± 2.47). As Hong Kong cattle have a wide variety of phenotypes (Barbato et al., 2020; Perroux et al., 2025b), cattle are individually identifiable and distinguishable *via* physical appearance (such as body size, horn shape, and coat color), with identities known from previous observations (Hodgson et al., 2025b). The primary observer was familiar with identities from previous behavioral observations (Hodgson et al., 2025b), and the secondary observer was trained for identification in the days leading up to the study period in the field, with a photo catalogue and established individual descriptions.

### Behavioral observations and data collection

We used instantaneous scan sampling to record data between 13 March 2023 and 24 March 2023, with 128 sessions of 20 min duration (Altmann, 1974). Two trained observers positioned at varying locations relative to the group (at least 10–20 m away from the animals) recorded data (aided by binoculars) using mobile recording software ZooMonitor (Ross et al., 2022). A mean of 12.8 sessions were conducted per day (range 8–17, SD ± 3.16), with observations occurring between 09:00 and 17:00. Each 20-minute session consisted of 4 scans, sampling every 5 min with a total of 512 complete group scan samples. The data collection process was as follows:

1. At the start of each session in the field, a focal animal was selected using a random generator from a list of animals which were present and had not been a focal in the previous scan.
2. At each scan interval, we recorded following factors:
  a. The proximity of each animal in the herd relative to that focal animal (within 1 body length, within 3 body lengths (∼3 m), over 3 body lengths (over ∼3 m)) to the focal individual. The average body length of animals in this herd (from shoulder joint to tuber ischii) is approximately 1 m (Perroux et al., 2025b).
  b. The animal identities of the focal individual’s closest neighbors; nearest neighbor one (NN1), nearest neighbor two (NN2), and nearest neighbor three (NN3) (Rands, Muir & Terry, 2014; Hoyle, Miller & Rands, 2021).
  c. The behavioral activity and identities of all individuals present.
3. After fieldwork data collection was concluded, a ‘random conspecific’ animal was chosen as a control. The random conspecific was selected by randomly sampling an individual present during each scan that was not a near neighbor (NN1, NN2, NN2); this sampling was performed post-data collection using R to avoid any visual biases that may arise from random selection of animals in the field.

Each individual was observed for 5 s to determine their active behavior, with mutually exclusive behaviors classified into four categories (lying, standing (resting, ruminating or vigilant while standing), walking or foraging (grazing and browsing, including drinking)). All individuals within view of the observers and the other animals were recorded in the scan as part of the group. The mean number of sessions per focal animal was 7.53 (range 4–9, SD ± 1.17). In addition to the 128 focal sessions used for data analysis, sessions where cattle were externally disturbed by human or dog activity were terminated early and discarded from analysis (13 sessions), as well as sessions missing the behavior of all group members present or without the identity of the focal animal’s nearest neighbors due to visual obstruction (seven sessions). Each session occurred between 09:00 and 17:00, with observations condensed into 10 days to minimize any impact from changes in group composition, temperature or weather (Hoyle, Miller & Rands, 2021). Hong Kong weather in March is at the end of the dry season, with March 2023 having a mean monthly temperature of 21.3 degrees, total monthly rainfall of 70.3 mm, and 76% mean relative humidity (Hong Kong Observatory, 2024).

### Data preparation

To examine how synchrony was affected by dyadic social relationships, we used existing social relationships as a measure of dyadic affiliation, and calculated the dominance rank of each individual within the group (Hodgson et al., 2025b). These supporting behavioral interaction data were collected in addition to the behavioral synchrony data during an independent previous observation period between 17 August 2022 and 22 May 2023, with 44 h of all-occurrence sampling between 10:00 and 16:00 (Hodgson et al., 2025b). Although the synchrony data collection falls within this time period, no data were included in both observations. Supporting behavioral interaction data was collected by a familiar observer using mobile recording software ZooMonitor (Ross et al., 2022), recording allogrooming events (social grooming) and non-contact displacement behaviors (Hodgson et al., 2025b). All animals recorded in the synchrony data were also observed in the supporting behavioral interaction data. Allogrooming was defined as repeated licking by one animal on another animal’s body, and distinct bouts separated by a break of 10 s or longer (Laister et al., 2011; Hodgson et al., 2024). Dyadic affiliation was therefore defined as a binary measure of whether two animals had previously exchanged allogrooming. Dominance interactions were also recorded over this period, with non-contact displacement behaviors defined as the approach of one animal (the ‘winner’) causing another animal (the ‘loser’) to withdraw from their original position, and take at least three steps away from the performer (Christensen et al., 2002; Hodgson et al., 2024). These directed behaviors were transformed into a winner-loser matrix, and we used the randomized Elo-ranking methodology with 10,000 randomizations *via* R package ‘aniDom’ in R to calculate dominance scores for each individual (Sánchez-Tójar, Schroeder & Farine, 2018; Farine & Sánchez-Tójar, 2021). We then converted the dominance scores into ordinal dominance ranks from 1 to 17, with one indicating the highest-ranked individual in the group.

### Statistical analysis

Data calculations and analyses were performed using R version 4.4.3 (R Core Team, 2024), with data and code accessible *via* an online OSF repository (Hodgson et al., 2025a). Synchrony measures are sensitive to both group size and the number of activity categories, and the Fleiss Kappa coefficient is an intrarater reliability agreement measure which has been suggested to be the most suitable measure of synchrony, given its ability to control for expected synchrony level (Asher & Collins, 2012). This coefficient calculates whether group behavior is more or less synchronized than expected by chance, with the agreement coefficient *K* ranging from −1 (absence of synchrony) to 1 (total synchrony), with 0 representing if observed synchrony is equal to chance. However, we were unable to fully calculate the Fleiss Kappa coefficient due to fluctuating group membership between scans (Asher & Collins, 2012; Stoyan et al., 2018). Instead, we partially calculated a Fleiss Kappa coefficient of agreement for 33 scans in which all 17 animals were present, using function ‘*kappam.fleiss’* from package ‘irr’ (Gamer et al., 2005). In addition, for all scan observations we calculated group synchrony as the maximum percentage of animals simultaneously performing the same behavior per scan, *i.e.,* the percentage of animals performing the most-common behavior out of the four behaviors. We also calculated the mean degree of group synchrony across all observations. We also calculated the proportion of observations which were equal to or higher than thresholds of 70%, 90% and 100%, where all cattle were in agreement. These thresholds were chosen due to cattle being at least 70% synchronous on the majority of occasions in previous research (Stoye, Porter & Stamp Dawkins, 2012).

To ensure that any results were not linked to occurrences where cattle were engaging in high foraging activity, we also performed proceeding models ii–iii on a subset of the larger dataset. This subset only included scans where less than 80% of animals were foraging (258 scans out of 512, 50.39%). This threshold was determined by visually inspecting a histogram of the percentage of animals foraging per scan (Table S1) while also excluding data which was half a standard deviation (17.44%) above the mean foraging percentage (64.59%). This excluded 254 scans out of 512 (Table S1; 49.61%) where animals were foraging at or above the 80% threshold. When investigating the effect of spatial proximity on synchrony (ii), we only included sessions where all four scans were below the 80% threshold, and removed any sessions where animals were foraging at or above the 80% threshold in one or more scan. This resulted in 45 sessions out of 128 (35.16%) included in the final model. All proceeding models were tested to ensure they fit model assumptions using packages ‘DHARMa’ and ‘performance’ (Hartig & Lohse, 2022; Lüdecke et al., 2024). Model significance was obtained through function ‘*drop1*” from package ‘stats’, with single-term deletions for all generalized linear models (GLMs) and generalized linear mixed-effects models (GLMMs), and obtained using Satterthwaite’s single-term deletions for linear mixed-effects models (LMMs).

#### Effect of sex and dominance on foraging (i)

To test whether sex and dominance affected foraging activity, we calculated the proportion of scans that each animal spent foraging; this was defined as the number of times that an animal was observed foraging divided by the individual’s total number of observations. We tested the effect of sex and dominance rank on the proportion of time spent foraging using a GLM (‘*glmmTMB’*, from R package ‘glmmTMB’) with a beta distribution and logit link (Brooks et al., 2023).

#### Effect of spatial proximity on synchrony (ii)

We investigated whether focal animals were more synchronized with their nearest neighbors or with another random animal in the group (the random conspecific). We calculated the proportion of scans in each session (*n*/4) that each type of animal (NN1, NN2, NN3, random conspecific) was synchronized with the focal animal (Rands, Muir & Terry, 2014; Hoyle, Miller & Rands, 2021). As the identities of the nearest neighbors to the focal animal were likely to change over the session, this model did not consider identity and neighbors were always the closest individuals. Synchrony therefore ranged from 0.00 (activity was never matched with focal’s activity) to 1.00 (activity was always matched with focal’s activity). We used a GLMM (‘*glmmTMB’*, R package ‘glmmTMB’), using the proportion of synchronized scans (number of synchronized scans/4) as a dependent variable and included the type of animal as an independent variable, with a logit link. Hour of observation and focal animal identity were fitted as random effects.

#### Effect of sex, dominance and affiliation on synchronization and proximity (iii)

To investigate whether synchrony between the focal animal and their nearest neighbor was affected by their sex, dyadic affiliation and dominance rank difference, we used each scan with a focal and their nearest neighbor (512 events). We classed each dyad as either same-sex (female–female or male-male) or different-sex (male–female). We assigned each dyad a binary measure of affiliation (whether the pair had previously exchanged allogrooming (1) or they had not (0)), and calculated the absolute dominance rank difference between their two ordinal ranks. We used a GLMM (function ‘*glmmTMB*’ from R package ‘glmmTMB’) with a binomial family and logit link, testing whether the predictor variables of dyadic sex combination (same-sex or different sex), previous affiliation (1 or 0), and dominance rank difference affected the binomial response of whether the two animals were synchronized (1) or not (0). The distance of the nearest neighbor from the focal (within one body length, within three body lengths, over three body lengths), the identities of both animals, and the hour of observation were fitted as random effects.

To test whether overall synchrony between two animals was related to their sex, dyadic affiliation and dominance rank differences, we recorded all possible dyadic pairs of animals (136 dyads). We then calculated dyadic synchrony as the proportion of scans where both animals were present and performing the same behavior (Šárová, Špinka & Panamá, 2007; Hauschildt & Gerken, 2015). We used a LMM (‘*glmmTMB’*, R package ‘glmmTMB’) to test whether dyadic sex combination (same-sex or different sex), previous affiliation (1 or 0), and absolute dominance rank difference affected the proportion of scans synchronized, with both cattle identities entered as random factors (Šárová, Špinka & Panamá, 2007). For comparison, we calculated the dyadic expected degree of synchronization which would occur by two animals (A and B) behaving independently (Šárová, Špinka & Panamá, 2007). This was calculated as:

\begin{eqnarray*}\sum _{1}^{x} \left( {P}_{A}\times {P}_{B} \right) \end{eqnarray*}

where *x* was the number of behavioral categories (4; lying, standing, foraging, walking), and *P* was the proportion of scans that A and B spent active in *x* behavioral category. We used a paired *t*-test to test whether dyads differed in their expected synchrony *versus* their observed synchrony, *via* function ‘*t.test*’ in package ‘stats’.

To test whether sex, dominance and affiliation affected the likelihood of proximity between each dyad, we used body length distance between the focal animal of each session and all other animals. We calculated the likelihood of a dyad being in close proximity as the number of times that both animals were recorded as within three body lengths of each other, divided by the total number of times that they were both present and one was a focal animal. We then ran a LMM (‘*glmmTMB’*, R package ‘glmmTMB’) to test whether the fixed variables of dyadic sex combination (same-sex or different sex), previous affiliation (1 or 0), and absolute dominance rank difference affected the likelihood of being in close proximity, with both animal’s identities fitted as random factors.

## Results

### Synchronization and sex differences in foraging (i)

Feral cattle were highly synchronized, with a mean maximum group synchrony degree of 79.89% out of 512 scans (range 28.57%–100%, SD ± 19.00), spending 69.92% of scans at or above the 70% synchrony threshold (including the 90% and 100% data). Cattle spent 40.43% of scans synchronized at or over the 90% threshold level, and were 100% in synchrony for 26.95% of scans. When only considering sessions where all four scans were below the 80% foraging activity threshold, mean maximum group synchrony was 66.88% (range 28.57%–100%, SD ± 17.63). The Fleiss Kappa coefficient of agreement (*K*) for 17 cattle with 33 observations and four categories of behavior was 0.56, indicating moderate to substantial synchrony in cattle activity.

**Figure 1:**
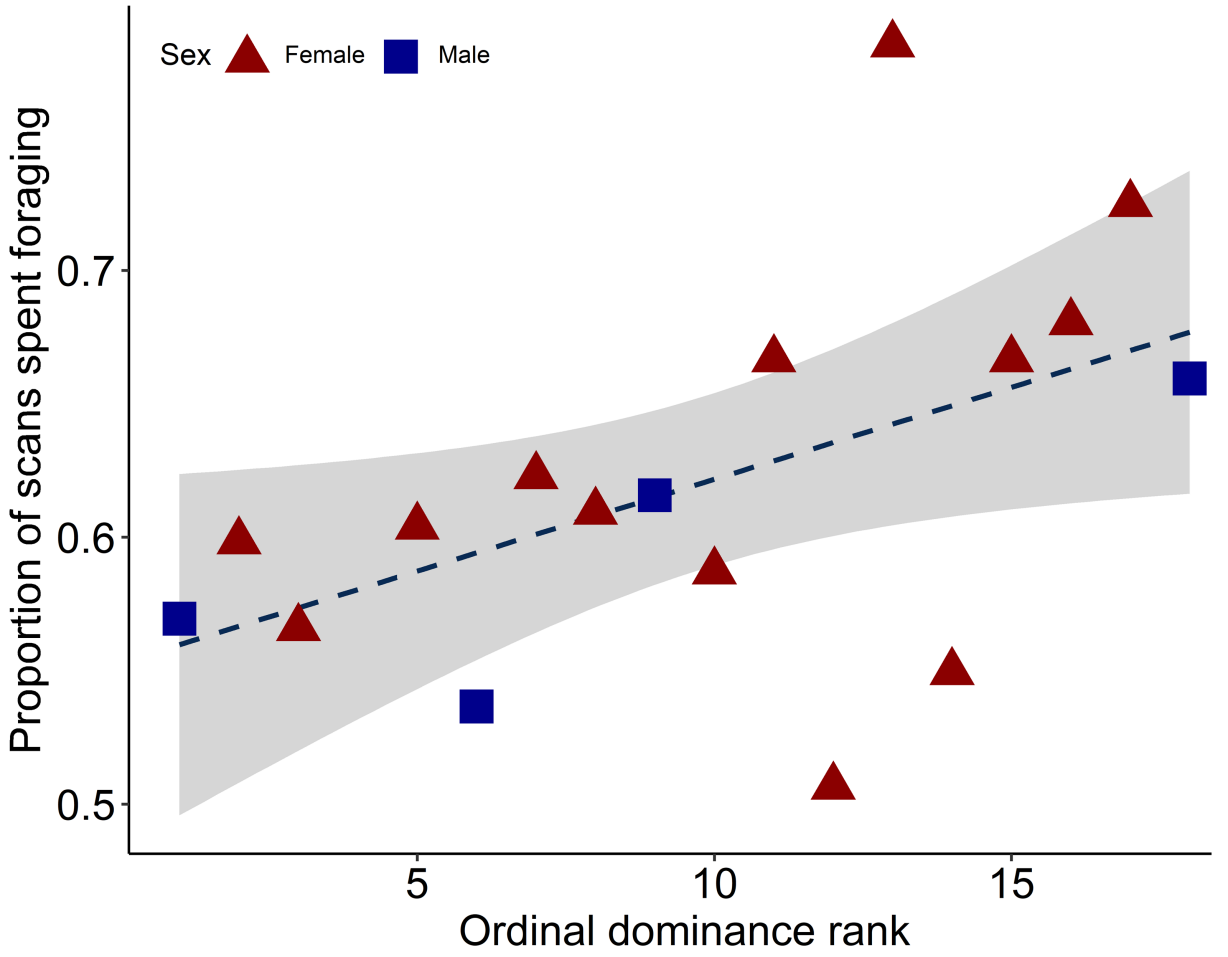
Individual proportion of scans spent foraging, relative to dominance rank. A rank of 1 represents the most dominant animal in the group. Female raw data points are represented by red triangles, male datapoints by blue squares. Trend line is from the linear model, grey areas represent 95% confidence intervals.

Rank affected the proportion of scans that an animal spent foraging, with lower-ranking animals spending more time foraging than higher-ranking animals (Fig. 1: GLM: estimate = 0.03, SE = 0.01, *z* = 2.41, *P* = 0.02). Sex had no effect on the proportion of scans spent foraging (GLM: estimate = −0.10, SE = 0.14, *z* =  − 0.67, *P* = 0.50). Individual cattle spent a mean of 62.1% of observed scans foraging (range 50.70%–78.50%, SD ± 7.01), 15.0% standing (range 8.21%–24.8%, SD ± 4.87), 11.5% lying (range 2.87%–17.1%, SD ± 3.99) and 11.4% walking (range 8.85%–15.00%, SD ± 1.93).

### Effect of spatial proximity on synchrony (ii)

**Figure 2:**
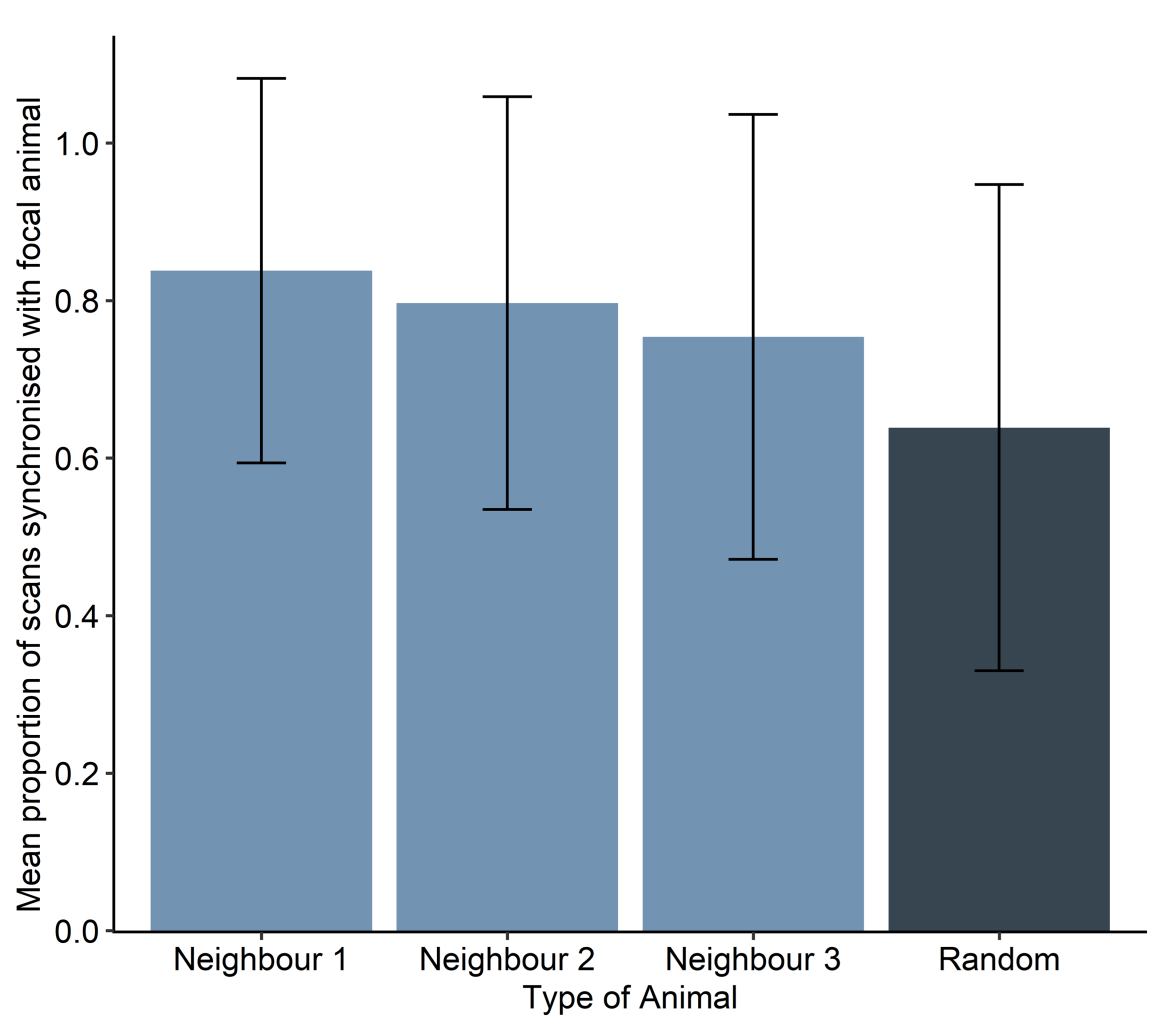
Mean proportion of scans synchronized with the focal individual per type of animal, from nearest neighbor 1 to 3, and a random conspecific. Error bars are the raw data standard deviation per group.

Focal animals were more synchronized with their nearest neighbor (NN1) than with a random conspecific (GLMM: estimate = 1.28, SE = 0.17, *z* = 7.55, *P* < 0.0001). Focal animals were also more synchronized with their second nearest neighbors (NN2, GLMM: estimate = 0.95, SE = 0.16, *z* = 5.89, *P* < 0.0001) and their third nearest neighbors (NN3, GLMM: estimate = 0.65, SE = 0.16, *z* = 4.17, *P* < 0.0001) than a random conspecific. Mean synchrony values between the focal animal and NN1 (0.84, SD ± 0.24), NN2 (0.80, SD ± 0.26) and NN3 (0.75, SD ± 0.28) were higher than between the focal animal and the random animal (0.65, SD ± 0.32; Fig. 2), with all values ranging from 0–1. These results remained consistent when excluding sessions with animals foraging at or above the 80% threshold in one or more scans, with focal animals being more synchronized with their nearest neighbor one (NN1, GLMM: estimate = 1.64, SE = 0.26, *z* = 6.41, *P* < 0.0001), two (NN2, GLMM: estimate = 1.19, SE = 0.24, *z* = 4.90, *P* < 0.0001) and three (NN3, GLMM: estimate = 0.79, SE = 0.24, *z* = 3.34, *P* = 0.0009) than the random conspecific.

### Effect of sex, dominance and affiliation on synchrony and proximity (iii)

Same-sex dyads were more synchronized than different-sex dyads (LMM: estimate = 1.63, SE = 0.65, *t* = 2.52, *P* = 0.01), with female–female and male-male dyads having a higher proportion of synchronization than female-male dyads. Dyadic synchrony was not affected by affiliation (LMM: estimate = 1.13, SE = 0.63, *t* = 1.79, *P* = 0.07) nor dominance rank difference (LMM: estimate = −0.11, SE = 0.07, *t* =  − 1.60, *P* = 0.11). However, when excluding scans with animals foraging at or above the 80% threshold, synchrony was affected by all three factors. Dyadic synchrony was higher between same-sex dyads (LMM: estimate = 2.51, SE = 1.00, *t* = 2.52, *P* = 0.01), had a negative association with rank difference (LMM: estimate = −0.26, SE = 0.11, *t* =  − 2.47, *P* = 0.01), and had a positive association with affiliation (LMM: estimate = 2.05, SE = 0.99, *t* = 2.08, *P* = 0.04). The mean observed proportion of dyadic synchrony was 67.98% (range 55.17%–78.78%, SD ± 4.78), with an expected mean dyadic synchrony of 43.33% (range 35.50%–58.79%, SD ± 4.61), and dyads were more synchronized than the expected distribution of dyadic synchrony if animals were behaving independently (paired-samples t-test: *t*_135_ = 55.72, *P* < 0.001).

The likelihood of a dyad being in close proximity (within 3 body lengths) was not related to the sex combination of the dyad (LMM: estimate = 0.76, SE = 1.60, *t* = 0.47, *P* = 0.64), affiliation (LMM: estimate = 0.63, SE = 1.66, *t* = 0.38, *P* = 0.70), or dominance rank difference (LMM: estimate = −0.02, SE = 0.17, *t* =  − 0.13, *P* = 0.90). These results were consistent when scans with high foraging activity were removed, with dyadic proximity likelihood unaffected by sex combination (LMM: estimate = 0.86, SE = 2.59, *t* = 0.33, *P* = 0.74), previous affiliation (LMM: estimate = 1.37, SE = 2.75, *t* = 0.50, *P* = 0.62), or dominance rank difference (LMM: estimate = −0.05, SE = 0.29, *t* =  − .19, *P* = 0.85). Likewise, the synchrony between a focal animal and its nearest neighbor was not affected by dyadic sex combination (GLMM: estimate = −0.36, SE = 0.38, *z* =  − .93, *P* = 0.35), previous affiliation (GLMM: estimate = −0.24, SE = 0.35, *z* =  − 0.70, *P* = 0.48), or dominance rank difference (GLMM: estimate = −0.01, SE = 0.04, *z* =  − .36, *P* = 0.72). These results were consistent when scans with high foraging activity were removed, with synchrony between a focal animal and its nearest neighbor unaffected by dyadic sex combination (GLMM: estimate = −0.29, SE = 0.47, *z* =  − 0.61, *P* = 0.54), previous affiliation (GLMM: estimate = 0.13, SE = 0.48, *z* = 0.30, *P* = 0.76), or dominance rank difference (GLMM: estimate = −0.03, SE = 0.05, *z* =  − 0.58, *P* = 0.56).

## Discussion

Although behavioral synchronization plays a vital role in movement coordination and collective behavior, it is often difficult to determine the relationships between proximity, individual characteristics and synchrony in wild ungulate populations (Duranton & Gaunet, 2016; Amichay et al., 2024). Using feral cattle with known social relationships, we determined that sex affects the likelihood of synchrony, with same-sex dyads having higher synchrony than different-sex dyads. Our results also demonstrate that proximity affects the likelihood of synchrony. With a mean group synchrony of approximately 80%, matching similar values in sheep (Gautrais et al., 2007) and farm cattle (Stoye, Porter & Stamp Dawkins, 2012), dyads were more synchronized than expected by chance. Rank difference and previous affiliation played a role in dyadic synchronization after the exclusion of scans with high foraging activity, but surprisingly we did not find any effect of affiliation or dominance rank difference on the likelihood of a dyad being in close proximity or synchrony between nearest neighbors. Although we found that higher-ranking animals spent less time foraging, sex did not affect foraging activity. Synchronization may come with varying costs to the individual, and our results highlight how underlying processes can affect movement in group-living mixed-sex ungulates (Ruckstuhl & Neuhaus, 2002; Bonenfant et al., 2004).

### Rank and sex affect foraging activity and dyadic synchrony

Our first hypothesis (i) was partially supported as subordinate animals spent more time foraging than dominant animals, but there was surprisingly no difference in time spent foraging between males and females. Lower-ranking animals are expected to consume lower-energy resources when higher-ranking animals are present, requiring them to spend more time foraging to compensate (Rands et al., 2006). Dominant feral cattle may monopolize resources (as found similarly in a range of social taxa, from vulturine guineafowl (*Acryllium vulturinum*; Papageorgiou & Farine, 2020) to chimpanzees (*Pan troglodytes*; O’Malley et al., 2016), and disturb subordinate animals when foraging, with subordinates being more likely to leave patches of food before they are finished (Ward & Webster, 2016). Feral cattle in Hong Kong have linear, steep and stable hierarchies (Hodgson et al., 2025b), with our results suggesting that dominant feral cattle could use their status to monopolize resources and thus obtain fitness-related benefits from their hierarchy position. Contrary to our predictions, sex did not affect foraging activity. Although feral cattle in Hong Kong are sexually dimorphic in body size and males are larger than females, this dimorphism is moderate, and unknown factors such as health status or age may also obscure sex differences (Perroux et al., 2025b). The effect of individual traits on food access is expected to be context-dependent (Kidjo et al., 2016), and sex differences in this group may also have been obscured by the sex ratio (four males to 13 females); further investigation into mixed-sex herds with varying sex ratios could help elucidate any effect of sex on foraging strategy. Larger rumens in male ungulates are expected to allow them to consume abundant lower-quality forage, with smaller-bodied females having larger requirements for high-quality forage, especially during reproduction (Ruckstuhl, 1998; Barboza & Bowyer, 2000). It is difficult to determine whether male and female cattle ate similar quality food items as we did not record the types of vegetation that individuals were consuming. However, individuals were observed eating similar types of vegetation, and it is unlikely animals were selecting different plants within the same area.

We found that same-sex dyads were more synchronized than animals of different sexes, partially supporting our third hypothesis (iii) and highlighting how synchronization may help to shape the evolution of sexual segregation. Same-sex synchronization has been found in other ungulates (*e.g.*, sheep, *Ovis aries*; Michelena et al., 2006), with red deer being more synchronized in single-sex groups than mixed-sex groups (Bonenfant et al., 2004). As we did not find any sex differences in foraging frequency, this indicates that activity differences may be temporal, with male and female feral cattle switching activities to synchronize with others of the same-sex, but ultimately performing the behavior at the same rate (Ruckstuhl & Kokko, 2002; Sueur & Deneubourg, 2011). It is not unusual for other ungulates to lack sexual differences in activity; for example, male and female mule deer (*Odocoileus hemionus*) are more efficient when foraging together (Bowyer & Kie, 2004). Interestingly, we find that rank difference and affiliation only affected dyadic synchrony when observations of high levels of synchronized foraging activity were excluded. This suggests that the effect of rank and dominance on synchronization may be activity-specific. Social factors may play a larger role after accounting for environmental drivers (such as resource heterogeneity), or in other kinds of coordinated movement, such as leadership (Krueger et al., 2014; Sueur et al., 2018). Other social factors (such as male harassment, kinship and familiarity) may differentially affect dyads, with familiarity increasing synchronization in farmed cattle (Gygax, Neisen & Wechsler, 2010), and shared stressful experiences leading to closer proximity in sheep (Keshavarzi et al., 2023). These dyadic relationships may mediate an animal’s response to neighbors in addition to sex, with species-specific differences helping to identify internal and external drivers of synchronization.

### Proximity affects behavioral synchrony

Our second hypothesis (ii) was fully supported, as proximity affected behavioral synchrony and cattle were more synchronized with nearest neighbors than with another random conspecific in the group. Spatial proximity is likely to affect how animals orientate themselves, and is a common factor in synchrony in fallow deer (Hoyle, Miller & Rands, 2021), red deer (Rands, Muir & Terry, 2014) and farm cattle (Stoye, Porter & Stamp Dawkins, 2012). Conspecific behavior provides information on habitat features, with foraging individuals providing environmental cues about food location or quality, and resource availability may drive activity and preferred space use (Dall et al., 2005). However, other ungulate behaviors, such as vigilance, are also affected by proximity and familiarity, with bighorn sheep (*Ovis canadensis*) being more likely to mimic vigilance in neighboring sheep which were spatially closer and more familiar (McDougall & Ruckstuhl, 2018). Paying attention to and responding to the behavior of a neighbor may be a feedback mechanism allowing the group to transmit information through behavioral contagion and stay cohesive; mimicry in activity thus leads to the maintenance of close proximity and subsequently further synchronization (Lakin et al., 2003; McDougall & Ruckstuhl, 2018).

### Limitations

All observations were mixed-sex, which unfortunately did not allow for us to investigate whether single-sex group synchrony differed from mixed-sex group synchrony. As individual cattle sterilization status is unknown in this population, our results may have been affected by variation in reproductive status (Pinkham et al., 2022; Agriculture, Fisheries and Conservation Department of Hong Kong (AFCD), 2024). Although assumed to be a breeding population due to calf presence, we cannot exclude that changes in hormonal status may have affected our results, as castration is known to affect behavior in male cattle (Price et al., 2003). Without individual level sterilization status, it is difficult to assess the impact of reproductive differences on behavior. Behavioral synchrony can also be driven by habitat heterogeneity, and the distribution of food resources within an area. Given that we were unable to quantify the forage quality and quantity consumed by each animal, we cannot exclude that some level of synchrony is also affected by the environment. However, when excluding scans of high foraging activity, our results surrounding the effect of sex and proximity on synchrony remain consistent. As cattle also utilized the same areas for resting and grazing (G Hodgson, 2023, pers. comm), this suggests that the role of sex and proximity in synchronization is independent of similar environmental drivers, such as the distribution of resources.

The lack of relationship between proximity and any sociodemographic factor is unexpected, as well as our result that overall synchrony between nearest neighbors was not affected by sex, dominance nor affiliative relationship. Our proximity-related results may be limited by our ability to only include inter-individual distances to focal animals. As the likelihood of synchrony and movement may depend on spatial position within the herd, we suggest that further work investigates synchrony relative to herd-level proximities to disentangle effects of nearest-neighbor behavior and within-group centrality (Morrell, Ruxton & James, 2011). Finer-scale individual differences may also affect proximity and animals may only change their behavior when close neighbors move out of sight. Dyadic proximity is often determined through shared motivation, and not related to the relationships between individuals (Asher & Collins, 2012). Instead of social facilitation, activity may be coordinated through collective responses to environmental cues, resulting in the coincidental alignment of behaviors (Asher & Collins, 2012; Camazine et al., 2020). Animals may act independently due to the influence of ecological stimuli, such as a high-quality foraging patch or an environment with reduced predation risk to rest in; due to the lack of large natural predators in Hong Kong, the latter may be unlikely in this study’s population (Dudgeon & Corlett, 1994). Cattle foraging behavior has previously not been associated with vegetation characteristics (Gabrieli & Malkinson, 2024), with social associations being random in cattle when grazing in smaller groups (Stephenson, Bailey & Jensen, 2016). We are ultimately unable to exclude the possibility of a common attractor (such as forage quality) affecting the self-organization of individuals and the likelihood of synchrony between individuals (Camazine et al., 2020). However, by removing scans where animals were foraging at or above the 80% threshold and finding that sociodemographic factors affected synchrony, we suggest that both social facilitation and environmental factors may play a role in ungulate synchronization. Without foraging and nutritional requirement information, these nuances are difficult to determine in wild-living groups, and we suggest further investigation into the differences between true coupling through social facilitation and independent responses in cattle outside of foraging.

## Conclusions

We investigated whether differences in individual sex, social phenotype and proximity affected foraging activity, synchronization and the likelihood of proximity between two animals. In summary, our results find that differences in proximity and sex are likely to drive variation in activity synchronization, suggesting ungulate synchronization when living in mixed-sex groups is biased towards animals in close spatial proximity and those of the same-sex. Although external motivation can drive some level of synchrony in activity, social attraction as a mimetic behavior could be an efficient individual-level mechanism driving cohesive moment in group-living animals. Understanding these trade-offs and the underlying processes that affect collective movement therefore allows us to comprehend different scales of behavior in group-living social animals, and our results highlight the importance of individual activity in collective ungulate decision making.

##  Supplemental Information

10.7717/peerj.21331/supp-1 Supplemental Information 1 R Code

10.7717/peerj.21331/supp-2 Supplemental Information 2 Summary of percentage of animals foraging per behavioral scan with cumulative numbers above the lower percentage threshold

## Additional Information and Declarations

### Data Availability

The following information was supplied regarding data availability: The data is available at OSF: George M.W. Hodgson, Kate J. Flay, Tania A. Perroux, and Alan G. McElligott. 2026. “Spatial Proximity and Dyadic Social Relationships Affect Ungulate Behavioral Synchrony.” OSF. March 11. doi: 10.17605/OSF.IO/GWPB5.
