# Supplementary material for: Sex and spatial proximity affect ungulate behavioral synchrony
Source: PeerJ. 2026 May 20;14:e21331. doi: 10.7717/peerj.21331 (PMC13198196; doi:10.7717/peerj.21331)
Supplement: Supplemental Information 2 [file peerj-14-21331-s002.docx]

**Table S1: Summary of percentage of animals foraging per behavioral scan with cumulative numbers above the lower percentage threshold.**

| Foraging percentage range | Number of scans | Percentage of total scans | Cumulative scans above the lower percentage threshold | Cumulative percentage above the lower percentage threshold |
| --- | --- | --- | --- | --- |
| x = 100% | 126 | 24.61 | 126 | 24.61 |
| 90% ≤ x < 100% | 58 | 11.33 | 184 | 35.94 |
| 80% ≤ x < 90% | 70 | 13.67 | 254 | 49.61 |
| 70% ≤ x < 80% | 26 | 5.08 | 280 | 54.69 |
| 60% ≤ x < 70% | 34 | 6.64 | 314 | 61.33 |
| 50% ≤ x < 60% | 39 | 7.62 | 353 | 68.95 |
| 40% ≤ x < 50% | 20 | 3.91 | 373 | 72.85 |
| 30% ≤ x < 40% | 24 | 4.69 | 397 | 77.54 |
| 20% ≤ x < 30% | 25 | 4.88 | 422 | 82.42 |
| 10% ≤ x < 20% | 33 | 6.45 | 455 | 88.87 |
| 0% < x < 10% | 19 | 3.71 | 474 | 92.58 |
| x = 0% | 38 | 7.42 | 512 | 100.00 |
